# Axillary cutaneous metastasis of colon cancer with microsatellite instability-high and *BRAF* V600E mutation treated with curative-intent surgery: a case report

**DOI:** 10.1186/s40792-023-01780-y

**Published:** 2023-11-14

**Authors:** Daisuke Yamai, Yoshifumi Shimada, Hikaru Ozeki, Akio Matsumoto, Kaoru Abe, Yosuke Tajima, Mae Nakano, Hiroshi Ichikawa, Jun Sakata, Toshifumi Wakai

**Affiliations:** 1 Division of Digestive and General Surgery, Niigata University Graduate School of Medical and Dental Sciences, 1-757 Asahimachi-dori, Chuo-ku, Niigata, 951-8510 Japan; 2 Medical Genome Center, Niigata University Medical and Dental Hospital, 1-754 Asahimachi-dori, Chuo-ku, Niigata, Japan

**Keywords:** Cutaneous metastasis, Colon cancer, Microsatellite instability-high, *BRAF* V600E mutation

## Abstract

**Background:**

Colorectal cancer (CRC) metastasizes to various organs, while cutaneous metastases are rare. Although there have been several previous reports of axillary cutaneous metastases with other metastases of CRC, there has never been a report of axillary cutaneous metastasis of CRC that could be treated with curative-intent surgery.

**Case presentation:**

A 68-year-old female was diagnosed with an axillary cutaneous tumor and ascending colon cancer with invasion to the duodenum. Histopathological and immunohistochemical analysis revealed that the axillary cutaneous tumor showed adenocarcinoma and the same expression pattern for cytokeratin 7, cytokeratin 20, and CDX2 as the ascending colon cancer, and that proved to be *KRAS–NRAS* wild type, MSI-H, and with a *BRAF* V600E mutation. The patient underwent a two-stage resection with curative intent after receiving neoadjuvant chemotherapy which consisted of one cycle of modified FOLFOX6 followed by two cycles of FOLFOXIRI. During and after the two operations, the patient received a total of nine cycles of modified FOLFOX6 as adjuvant chemotherapy. Two years after the initial surgery, and 1 year and 8 months after the second surgery, the patient is alive without recurrence.

**Conclusions:**

To the best of our knowledge, this is the first report of axillary cutaneous metastasis of CRC with microsatellite instability-high and *BRAF* V600E mutation that could be treated with curative-intent surgery. It is important to recognize the presence of such cases for the accurate diagnosis and treatment of CRC with cutaneous metastasis.

## Background

Colorectal cancer (CRC) is the third most frequently diagnosed cancer worldwide [1]. CRC commonly metastasizes to the regional lymph nodes, lungs, liver, and peritoneum, while cutaneous metastases are very rare, with a frequency of 0.8–6.5% [2–5]. Cutaneous metastases of CRC have been reported to occur throughout the body, including the trunk, head, and extremities, but predominantly in areas close to the primary tumor, and the most frequent site is the abdominal skin [6, 7]. Cutaneous metastases are usually noted only in patients with advanced disease and they indicate a poor prognosis [8].

CRC has several biomarkers that have clinical significance in diagnosis, prognostic evaluation, and treatment prediction. Examples include genetic factors, such as *KRAS* and *NRAS* mutations, *BRAF* mutation, and microsatellite instability (MSI), the importance of which is well-established [9].

Herein, we present a very rare case of ascending colon cancer with axillary cutaneous metastasis that was microsatellite instability-high (MSI-H) and harboring a *BRAF* V600E mutation. Although there have been several previous reports of axillary cutaneous metastases with other metastases of CRC [10–12], there has never been a report of axillary cutaneous metastasis of CRC that could be treated with curative-intent surgery. In addition, there are few reports on the genetic background of cutaneous metastasis of CRC.

## Case presentation

**Fig. 1:**
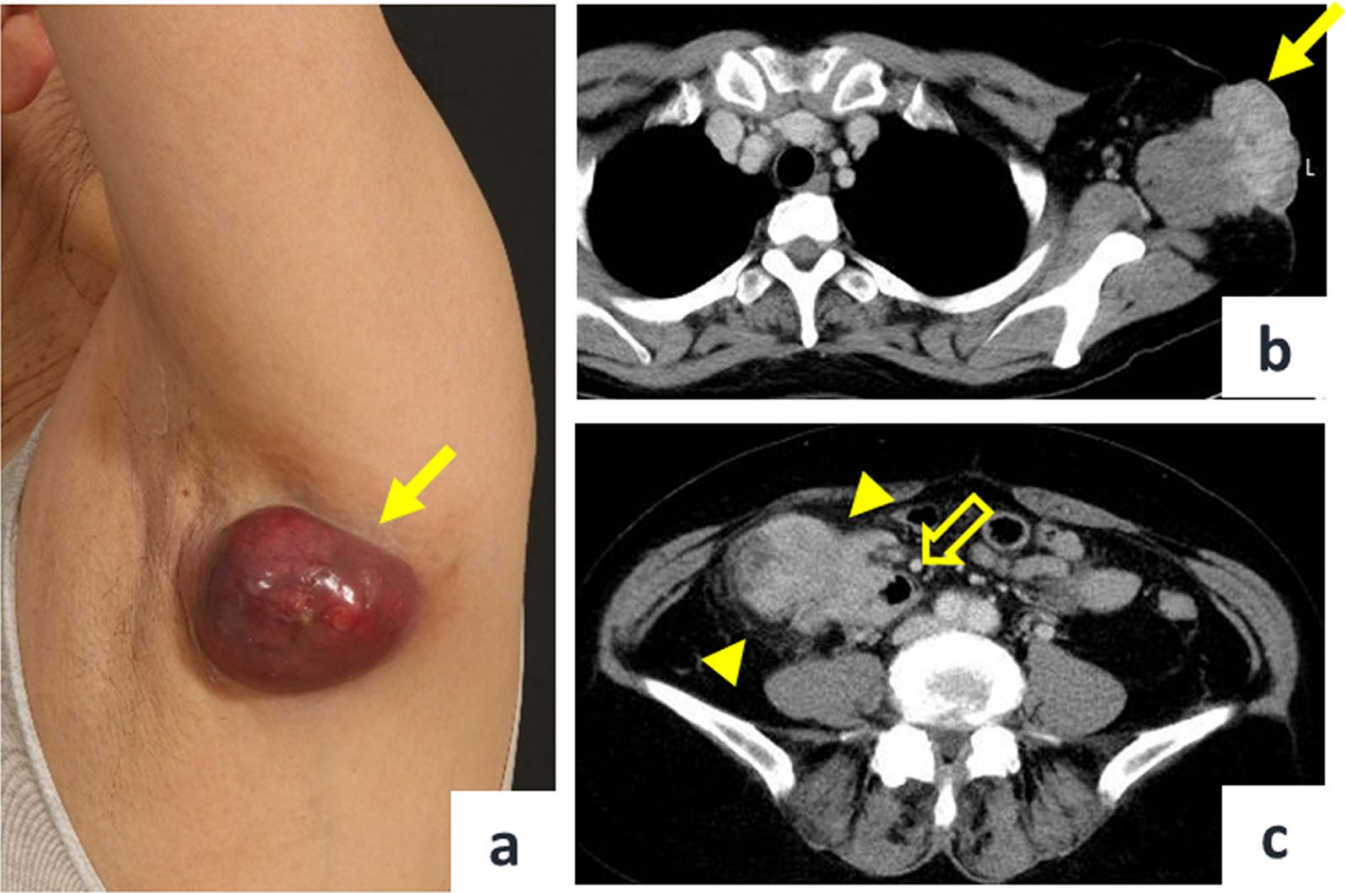
Pre-treatment clinical findings. **a** Cutaneous tumor measuring 5 cm in diameter located on the left axillary skin (solid arrow). **b** CT scan of the chest shows the cutaneous tumor in the left axilla with the invasion of the left teres major muscle (solid arrow). **c** CT scan of the abdomen and pelvis shows the circumferential enhancing wall thickening of the ascending colon (arrowheads) with invasion of the duodenum (open arrow)

**Fig. 2:**
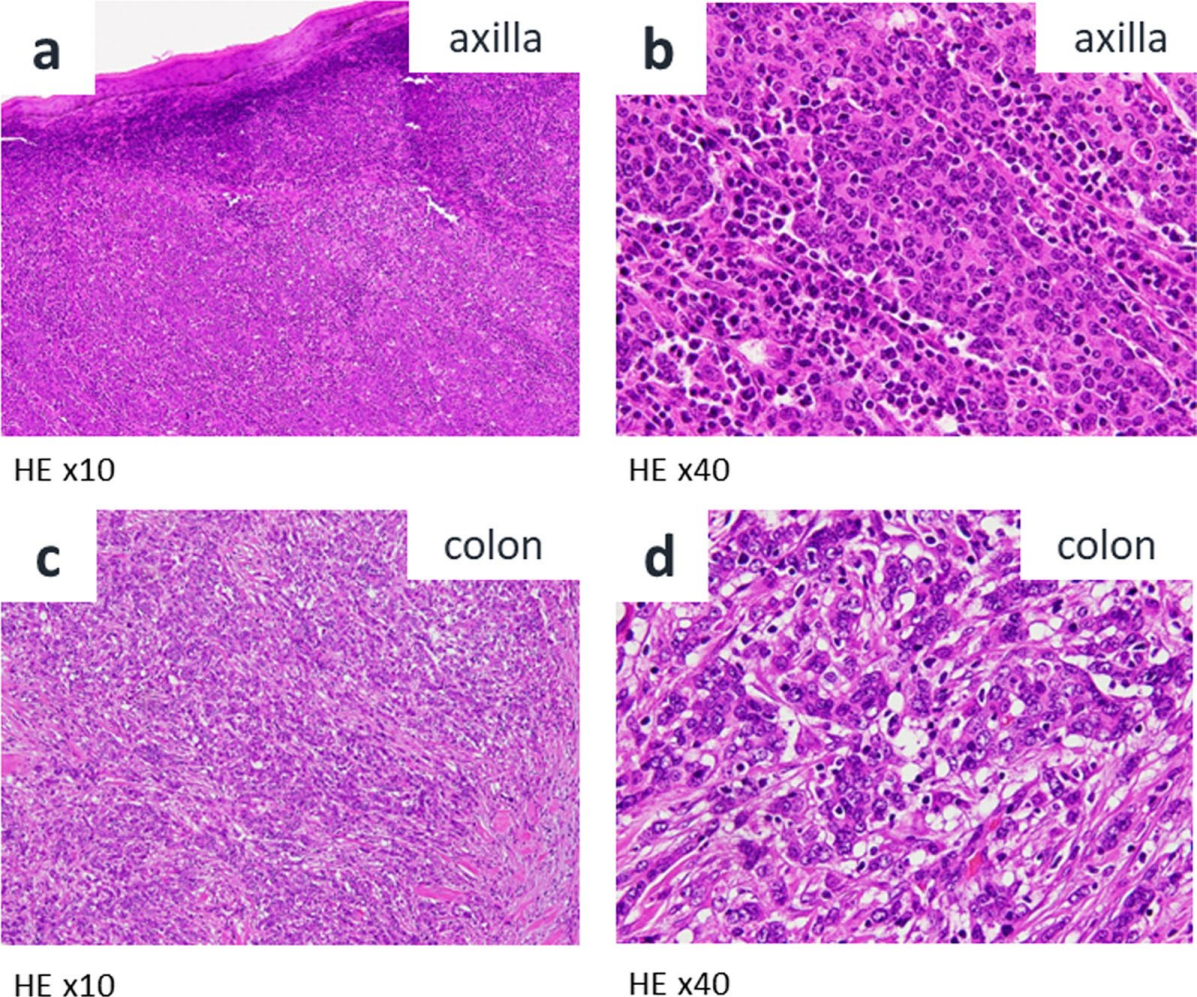
Histopathological examination. **a** Axillary cutaneous tumor consisted of poorly differentiated adenocarcinoma (hematoxylin–eosin, × 10). **b** Tumor-infiltrating lymphocytes are identified around tumor cells (hematoxylin–eosin, × 40). **c** Colon cancer consisted of poorly differentiated adenocarcinoma (hematoxylin–eosin, × 10). **d** Tumor-infiltrating lymphocytes are identified around tumor cells (hematoxylin–eosin, × 40)

**Fig. 3:**
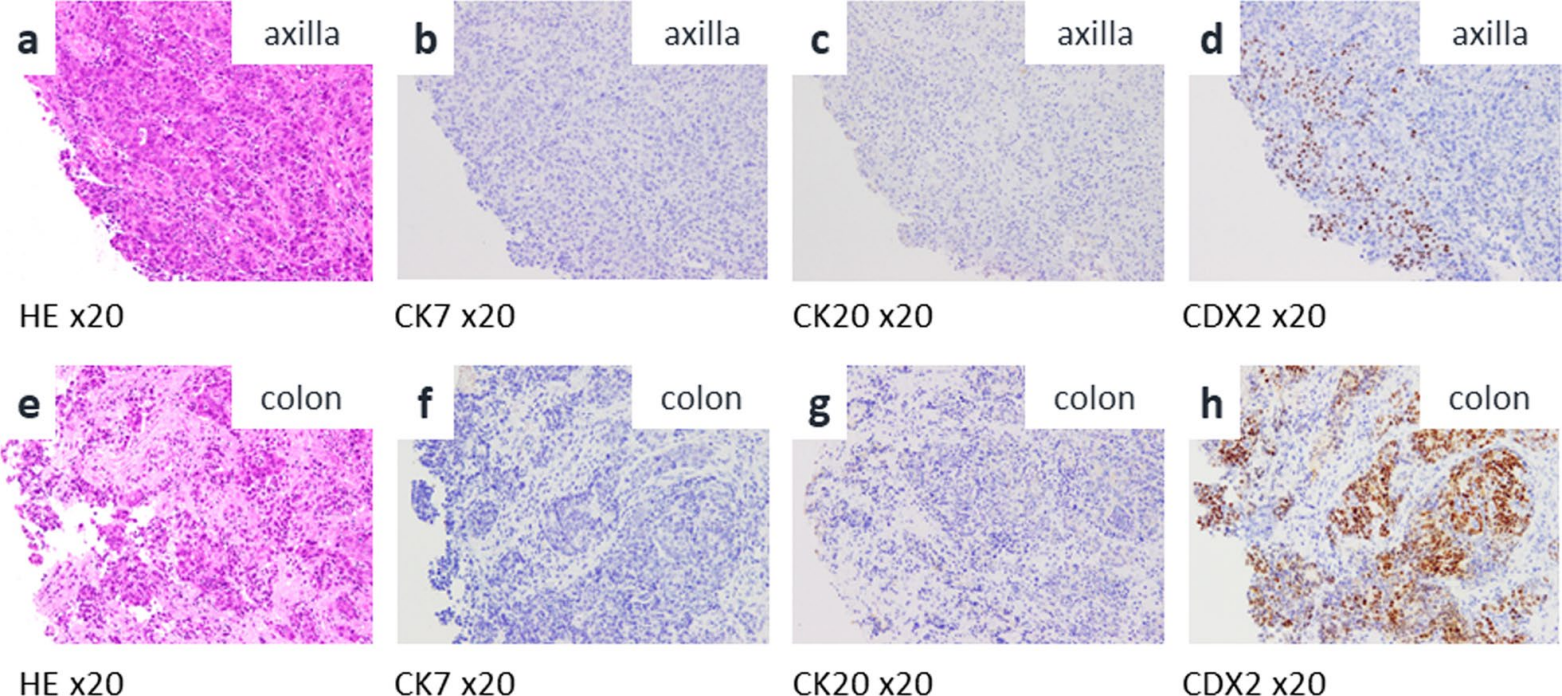
Immunohistochemistry (IHC) for CK7, CK20, and CDX2. Axillary cutaneous tumor and colon cancer were negative for CK7 and CK20, and positive for CDX2. **a**–**d** IHC analyses of the axillary cutaneous tumor (× 20). **e**–**h** IHC analyses of the colon cancer (× 20)

**Fig. 4:**
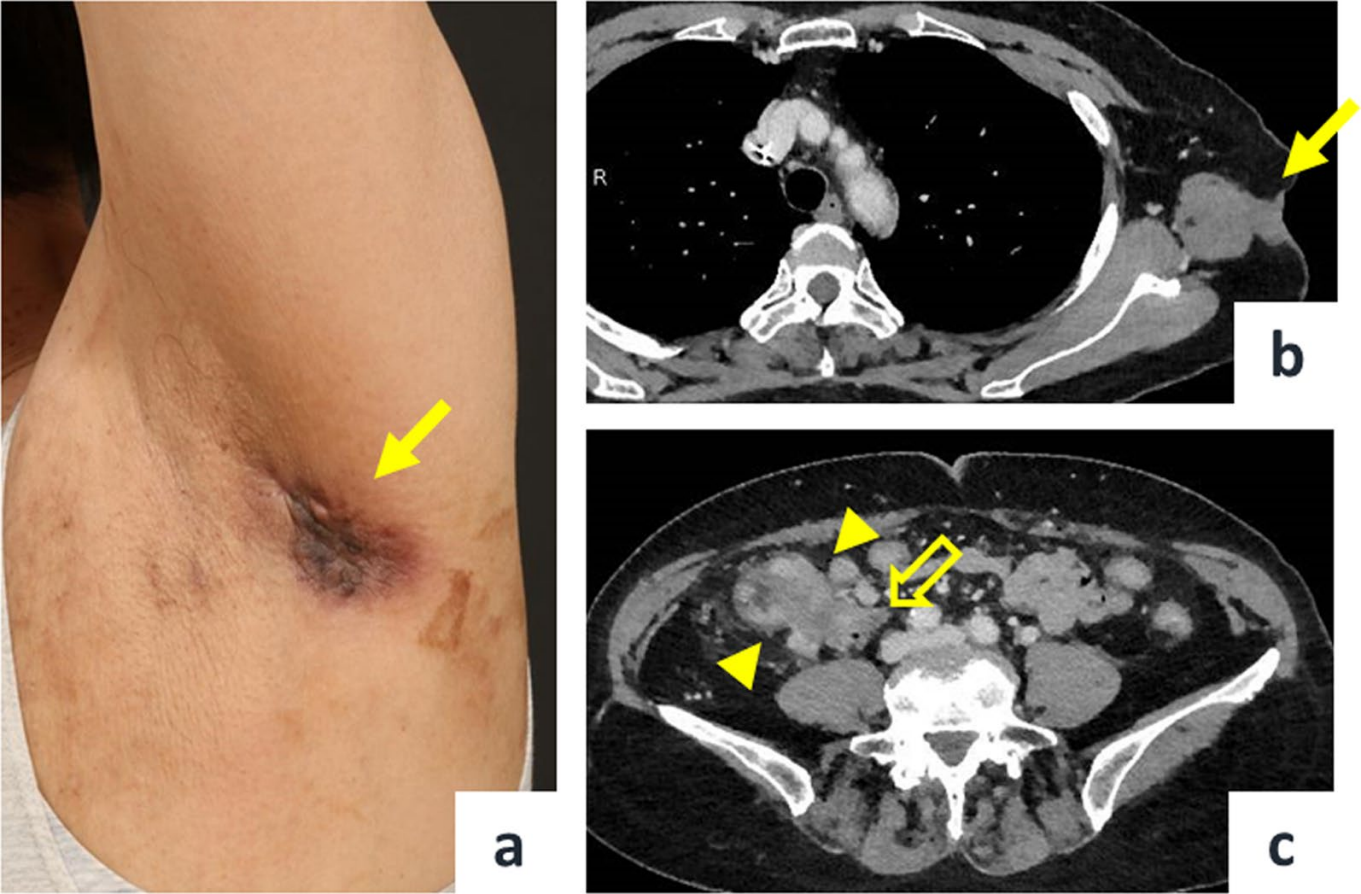
Post-chemotherapy clinical findings. **a** Axillary cutaneous tumor almost disappeared grossly (solid arrow). **b** CT scan of the chest shows the decreased size of the cutaneous tumor in the left axilla (solid arrow). **c** CT scan of the abdomen and pelvis shows the decreased size of the ascending colon cancer (arrowheads) with invasion of the duodenum (open arrow)

**Fig. 5:**
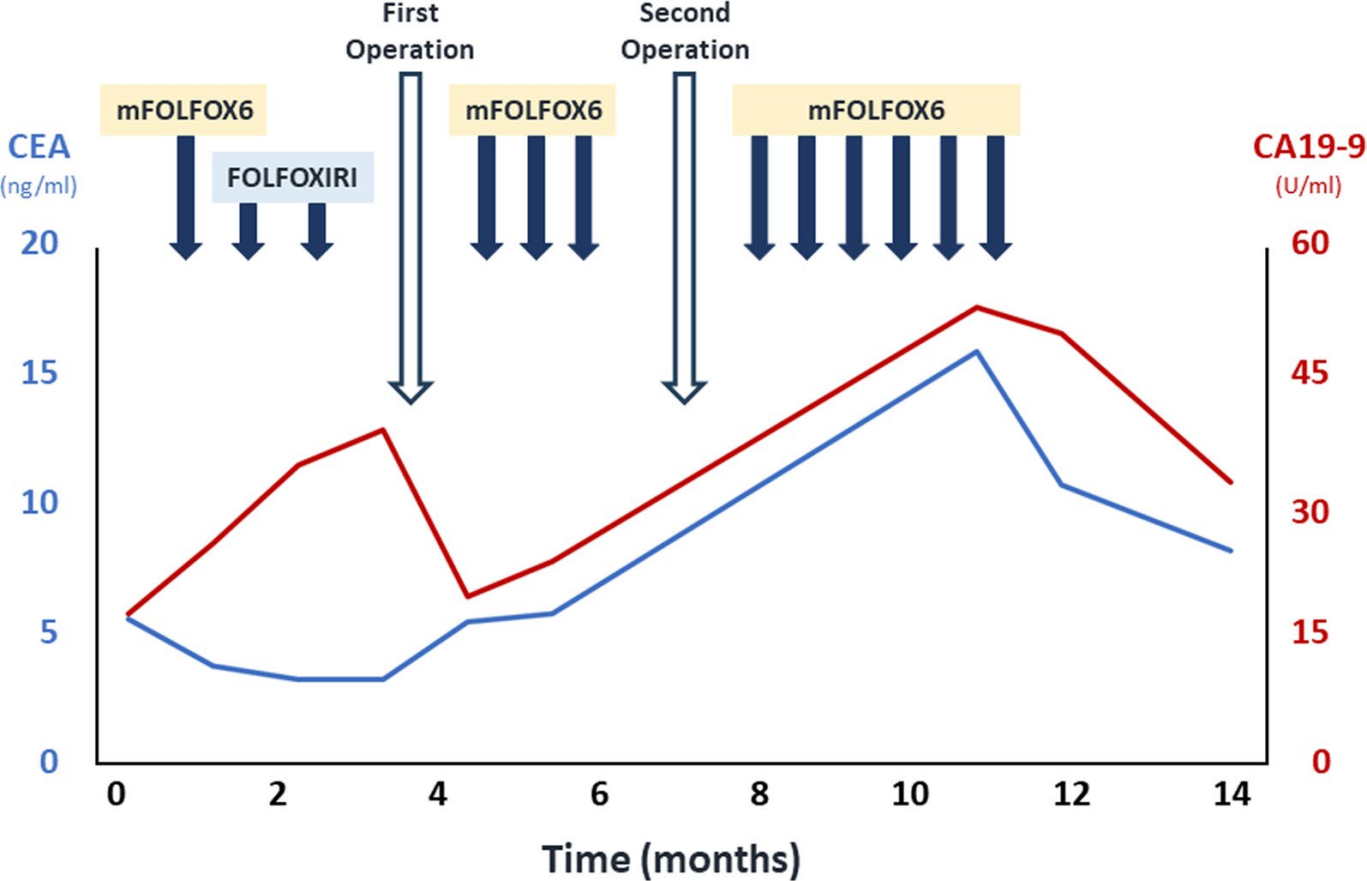
Clinical course of disease and changes in tumor marker values. First Operation means right hemicolectomy with lymph node dissection and partial resection of the duodenum with dissection of lymph nodes along the posterior pancreatoduodenal arcades, and Second Operation means resection of the axillary cutaneous tumor with axillary lymph node dissection

A 68-year-old female presented to the dermatologist with a chief complaint of a left axillary cutaneous tumor that had been growing for 2 months. She had no medical history, no surgical history, no family history of cancers or Lynch-syndrome-associated tumors, and was taking no medication. In addition, she had no history of smoking, did not drink alcohol, was 156.0 cm tall, weighed 52.3 kg, and had a body mass index of 21.5. On clinical examination, the tumor was 5 cm in diameter, elastic hard, dark red, non-tender, and with poor mobility at the left axilla (Fig. 1a). Laboratory examinations showed carcinoembryonic antigen and carbohydrate antigen 19-9 levels of 5.8 ng/ml and 18.0 U/ml, respectively. The patient underwent a cutaneous mass biopsy, and the histopathology showed poorly differentiated adenocarcinoma (Fig. 2a, b). Immunohistochemistry (IHC) revealed that the lesion was positive for anti-pan cytokeratin (CK) (AE1/AE3), and CDX2, and negative for CK7, CK20, ER, PgR, and TTF1. Based on these results, the cutaneous tumor was suspected to be a metastatic cutaneous cancer of gastrointestinal origin (Fig. 3a–d). Computed tomography (CT) scan of the thorax to the pelvis demonstrated the cutaneous tumor in the left axilla with invasion of the left teres major muscle (Fig. 1b), circumferential enhancing wall thickening of ascending colon with the invasion to the duodenum (Fig. 1c), and the presence of swollen left axillary and pericolic lymph, and of lymph nodes along the posterior pancreatoduodenal arcades. Mammography and breast ultrasonography were performed to rule out primary breast cancer, but no breast mass was found. Colonoscopy and esophagogastroduodenoscopy revealed a circumferential type 2 tumor of the ascending colon with invasion of the second part of the duodenum. Endoscopic biopsy revealed adenocarcinoma (Fig. 2c, d), and IHC showed the ascending colon cancer had the same expression pattern of CK7, CK20, and CDX2 as the cutaneous tumor (Fig. 3e–h). From the above, the left axillary cutaneous tumor was identified as a metastatic lesion from the ascending colon adenocarcinoma based on morphological similarity and immunohistochemical staining patterns. The clinical stage was Stage IVB (T4bN1aM1b) based on the American Joint Committee on Cancer, 8th Edition. Furthermore, mutational analysis found the tumor to be *KRAS–NRAS* wild type, *BRAF* V600E-mutant, and MSI-H. The patient received neoadjuvant chemotherapy which consisted of one cycle of modified FOLFOX6 (5-fluorouracil/leucovorin and oxaliplatin) followed by two cycles of FOLFOXIRI (5-fluorouracil/leucovorin, oxaliplatin, and irinotecan). Bevacizumab was not used because of concerns about fistula formation between the ascending colon cancer and the duodenum due to tumor invasion. Pembrolizumab was not used in view of the domestic regulatory approval situation at the time of treatment. After chemotherapy, the axillary cutaneous tumor was markedly reduced grossly (Fig. 4a), and CT scan showed that the primary tumor in the ascending colon and the axillary cutaneous tumor was reduced in size (Fig. 4b, c). The patient was scheduled for a two-stage resection with curative intent to allow intervals to confirm whether another metastasis to other organs and underwent primary resection, i.e., right hemicolectomy with lymph node dissection and partial resection of the duodenum with dissection of lymph nodes along the posterior pancreatoduodenal arcades, approximately 2 months after the initiation of chemotherapy. After the first surgery, the patient received three cycles of modified FOLFOX6, followed by a resection of the axillary cutaneous tumor with axillary lymph node dissection. Postoperative histopathological examination revealed that the ascending colon cancer consisted of poorly differentiated adenocarcinoma that invaded the duodenum with lymph node metastasis, and the axillary cutaneous tumor was almost certainly a metastasis of the colon cancer. After the second surgery, the patient received six courses of modified FOLFOX6 as adjuvant chemotherapy, and tumor markers decreased over time (Fig. 5). Two years after the initial surgery, and 1 year and 8 months after the second surgery, the patient is alive without recurrence.

## Discussion

Cutaneous metastases of internal malignancies are very rare with the incidences ranging from 0.7% to 5.0%, the most frequent sources being breast and lung cancers [6, 13–16]. It is uncommon for CRC to develop cutaneous metastases, which typically occur in patients with widely disseminated disease and frequently have a poor prognosis [8]. The average survival of patients after diagnosis of cutaneous metastasis from CRC is 18 months, ranging from approximately 1–34 months [6, 13, 17]. The most frequent cutaneous sites of CRC metastasis are generally close to the primary tumor site, such as the abdominal wall, umbilical region (Sister Mary Joseph nodule), or operative scar, but distant metastases can also occur [6, 17, 18]. Other cutaneous sites include the pelvis, back, thorax, extremities, head, and neck [6, 16, 19]. Several mechanisms of cutaneous metastases have been postulated, including lymphatic or hematogenous spread, direct extension of tumor, surgical implantation, and spread along the embryonal remnants, such as the urachus [4, 7]. Cutaneous metastasis often presents simultaneously with liver, lung, or peritoneal metastases, thus cases in which cutaneous metastases appear as solitary lesions are extremely rare [16, 20]. Therefore, previous cases of CRC with axillary cutaneous metastases have also had metastases to other sites synchronously, and there has never been a report of axillary cutaneous metastasis of CRC that could be treated with curative-intent surgery [10–12]. To the best of our knowledge, this is the first report of axillary cutaneous metastasis of CRC that could be radically resected.

MSI-H status in CRC has significant clinical implications. MSI-H tumors are characterized by widespread MSI, typically caused by defects in DNA mismatch-repair genes. The overall percentage of MSI-H in CRC is 12–16% in Western studies, and 6–7% in Japanese studies [21]. MSI-H is associated with right-sided colon carcinomas, lymphocytic infiltrate, and a poorly differentiated, mucinous, or signet ring appearance [22]. Moreover, MSI-H is commonly observed in Lynch syndrome, an inherited genetic condition predisposing individuals to various cancers, including CRC [23]. MSI-H is reported to be a favorable prognostic factor in Stage II/III CRC, but it is reported to be a poor prognostic factor in metastatic CRC (mCRC), based on an integrated analysis of several clinical trials [24, 25]. In mCRC, the prevalence of MSI-H is low, and MSI-H CRC has reduced metastatic potential [26]. MSI-H CRC tends to develop peritoneal metastases, and extraperitoneal metastases, such as liver and lung metastases, are less frequent than in microsatellite stable (MSS) CRC [27, 28]. The host immune response to tumors with lymphocytic infiltration, such as tumor-infiltrating lymphocyte and Crohn’s-like lymphoid reaction, in MSI-H CRC is thought to be associated with better prognosis and reduced distant metastasis [29, 30].

Methylation of the *MLH1* promoter region which is typically seen in sporadic MSI-H CRC, but not in Lynch syndrome, is strongly associated with the *BRAF* V600E mutation [31, 32]. *BRAF* mutations, particularly the V600E mutation, may be detected in approximately 12% of patients with CRC, and are associated with poor prognosis [33]. In Japan, the frequency of *BRAF* mutations in CRC is approximately 5%, which is lower than in Western countries [34]. When limited to patients with MSI-H CRC, *BRAF* mutations increase in frequency to 30–34.6% [25, 35]. MSI status and *BRAF* mutation are significant interacting prognostic factors. Although *BRAF* mutation is associated with poor prognosis, the presence of MSI may attenuate its adverse effect. *BRAF*-mutant CRC has a worse prognosis than *BRAF* wild type in patients with MSI-H CRC, but both groups have a better prognosis than patients with MSS CRC without *BRAF* mutation. Similarly, *BRAF* mutation is a poor prognostic factor in patients with MSS CRC, and MSS CRC with *BRAF* mutation has the worst prognosis overall [36, 37]. American Joint Committee on Cancer eighth edition and several previous studies have reported that the presence of *BRAF* mutation in MSS CRC was strongly associated with poor prognosis, whereas the presence of *BRAF* mutation in MSI-H CRC had a limited prognostic effect [38, 39]. *BRAF*-mutant CRC had significantly higher rates of peritoneal metastases and distant LN metastases, and lower rates of lung metastases compared to *BRAF* wild type [35]. Christensen et al*.* reported that the *BRAF* V600E mutation is associated with an increased risk of cutaneous metastases [40]. Although a few cases of cutaneous metastasis of *BRAF*-mutant CRC have been reported in fact, the number is small, and further accumulation of cases is needed to explore the relationship between *BRAF* mutation and cutaneous metastasis [12, 20, 41]. Yunoki et al*.* reported the first case of cutaneous metastasis of CRC that was MSI-H and *BRAF* V600E-mutant [42]. In that case, liver and lung metastases were observed simultaneously with the diagnosis of cutaneous metastasis of cecum cancer. Because the patient had unresectable distant metastases, systemic chemotherapy with CAPEOX (capecitabine plus oxaliplatin) plus bevacizumab was introduced after palliative resection of the primary tumor. Our case is very valuable in that radical resection of all lesions was achieved in MSI-H and *BRAF*-mutant CRC with cutaneous metastases, and no similar report has been published to date.

The ultimate diagnosis of cutaneous metastasis from CRC relies on histological evaluation. CK7, CK20, and CDX2 immunostaining are the most helpful markers to distinguish common malignancies, and CRC typically expresses CK20 and CDX2, but not CK7 [6, 7, 18]. CDX2 is a useful immunohistochemical marker of intestinal epithelium, and the presence of CDX2 in tumors of unknown origin raises the possibility of a gastrointestinal origin. In addition, loss of CDX2 expression has also been reported to be associated with *BRAF* mutation and MSI, and is a poor prognostic marker in patients with mCRC [43, 44]. Although clinicopathological features such as advanced tumor stage, vascular invasion, and poor differentiation have also been suggested to be associated with loss of CDX2, the association has been inconsistent between studies [45]. Hence, CDX2 is not currently used to predict prognosis and determine treatment strategies for CRC. Although the significance of CDX2 expression is not clear, it is possible that CDX2 expression is associated with favorable treatment response in our case.

Although standard treatment for cutaneous metastases of CRC has not been established, local excision is considered preferable for resectable cutaneous metastatic lesions [6]. Radiation therapy, systemic chemotherapy, and chemoradiation therapy are treatment options for unresectable lesions, but these options vary from case to case. In the KEYNOTE-177 trial, in previously untreated unresectable advanced recurrent MSI-H CRC, patients treated with pembrolizumab demonstrated a higher response rate and significantly prolonged progression-free survival compared to those treated with standard chemotherapy [46]. Furthermore, the efficacy of nivolumab monotherapy and the combination of ipilimumab, a CTLA-4 inhibitor, and nivolumab in previously treated patients with MSI-H mCRC, has been demonstrated in the CheckMate-142 trial [47, 48]. *BRAF*-mutant CRC is considered resistant to EGFR inhibition, and anti-EGFR antibody therapy does not increase the benefit of standard therapy in *BRAF*-mutant CRC patients [49]. The TRIBE study showed greater benefit from FOLFOXIRI plus bevacizumab compared with FOLFIRI plus bevacizumab in *BRAF*-mutant mCRC [50]. Based on these results, the use of FOLFOXIRI plus bevacizumab was considered the preferred option in *BRAF*-mutant tumors [51]. However, several subsequent trials did not show a survival benefit of the combination of FOLFOXIRI plus bevacizumab for *BRAF*-mutant mCRC compared to doublet plus bevacizumab, but did show a trend toward better response rates and progression-free survival [52]. In addition, based on the results of the BEACON trial, encorafenib plus cetuximab or encorafenib plus binimetinib plus cetuximab is recommended for *BRAF*-mutant mCRC treated with prior chemotherapy [53].

As described above, various biomarker-directed therapies have been established for CRC, resulting in a complex treatment system. However, no standard treatment has been established to date for *BRAF*-mutant MSI-H CRC. In our case, neoadjuvant chemotherapy was scheduled based on the assumption of curative-intent surgery. The patient received one cycle of modified FOLFOX6 to assess the patient's tolerability of chemotherapy and after confirming the absence of adverse events, chemotherapy was switched to FOLFOXIRI. This approach was based on several reports showing that triplet chemotherapy significantly improved response and survival rates compared to doublet chemotherapy in the treatment of mCRC [50–52, 54]. Bevacizumab was not administered because of concerns about the side effect of fistula formation between the ascending colon cancer and the duodenum due to tumor invasion. Pembrolizumab was not administered in view of the domestic regulatory approval situation at the time of treatment. Although this currently may be the case, a policy of preoperative pembrolizumab induction followed by radical resection may be preferable, based on the latest findings and NCCN guidelines [9]. There is also no clear evidence regarding the efficacy of adjuvant chemotherapy after curative-intent surgery of distant metastases of mCRC; however, several randomized controlled trials have suggested the efficacy of perioperative adjuvant chemotherapy for liver metastases [55, 56]. The NCCN and ESMO guidelines also recommend oxaliplatin-based perioperative adjuvant chemotherapy for up to 6 months for CRC with resectable metastases [9, 57]. In our case, the tumor marker level tended to increase during postoperative adjuvant chemotherapy, but there were no findings suggestive of recurrence on positron-emission tomography and CT scan. Hence, modified FOLFOX6 was administered perioperatively for 6 months based on the efficacy of adjuvant chemotherapy shown for liver metastases of mCRC.

## Conclusion

We describe a patient with axillary cutaneous metastasis of CRC with MSI-H and *BRAF* V600E mutation. Although cutaneous metastases from CRC, especially in the axilla, are very rare, it is important to recognize the presence of such cases for accurate diagnosis and treatment. Further accumulation of cases are needed to explore more effective treatments for CRC with cutaneous metastasis.

## Data Availability

Not applicable.

## Abbreviations

CRC: Colorectal cancer
MSI-H: Microsatellite instability-high
MSI: Microsatellite instability
IHC: Immunohistochemistry
CK: Cytokeratin
CT: Computed tomography
mCRC: Metastatic colorectal cancer
MSS: Microsatellite stable
